# Survey of perioperative treatment in muscle-invasive bladder cancer using Japanese hospital-based claims database

**DOI:** 10.1186/s12894-025-01995-1

**Published:** 2025-11-24

**Authors:** Ayumi Yamazawa, Masami Tsuchiya, Shungo Imai, Keisuke Ikegami, Hayato Kizaki, Satoko Hori

**Affiliations:** Division of Drug Informatics, Keio University Faculty of Pharmacy, 1-5-30 Shibakoen, Minato-ku, Tokyo, 105-8512 Japan

**Keywords:** Muscle-invasive bladder cancer, Neoadjuvant chemotherapy, Radical cystectomy, Treatment patterns

## Abstract

**Purpose:**

Urothelial carcinoma (UC) is a common malignant tumor of the urinary tract, with bladder cancer as the most frequent type. Radical cystectomy (RC) is the standard treatment for muscle-invasive bladder cancer (MIBC), and perioperative chemotherapy is recommended in addition to RC in guidelines. However, no established evidence defines the optimal regimen and number of cycles, and treatment varies by institution. Therefore, this study aimed to clarify perioperative treatment patterns for MIBC using a hospital-based claims database.

**Methods:**

Patients with claims records for bladder cancer recorded during the case-identification window (2012–2020) were identified using the Medical Data Vision Co., Ltd. database. The database spans April 2008 to July 2021, enabling longitudinal assessment. Patient characteristics and prescribed perioperative chemotherapy regimens were analyzed. Factors associated with neoadjuvant chemotherapy (NAC) were examined using univariable analysis and an exploratory multivariable logistic regression model.

**Results:**

Among 6,723 patients, 78.5% were men, with a median diagnosis age of 71 years. The treatment groups included 2,324 patients who received NAC, 598 who received adjuvant chemotherapy (AC), 364 who received NAC and AC, and 3,437 who underwent RC-only. NAC utilization rate increased over time (*P* for trend < 0.0001). Gemcitabine plus cisplatin was the most frequently administered regimen. Univariable analysis revealed an association between clinical stage 3 or higher and the NAC administration. In contrast, older age and comorbidities were associated with non-administration of NAC. Furthermore, the hospital size and cancer hospital designation influenced NAC administration.

**Conclusion:**

Perioperative chemotherapy utilization for MIBC has increased annually. Patient characteristics and treatment facility factors influence NAC administration.

**Supplementary Information:**

The online version contains supplementary material available at 10.1186/s12894-025-01995-1.

## Background

Urothelial carcinoma (UC) is a malignant tumor of the urinary tract (renal pelvis, ureters, bladder, and urethra), with bladder cancer being the most common type. Bladder cancer is classified into three clinical stages: non-muscle invasive bladder cancer (NMIBC), muscle-invasive bladder cancer (MIBC), and metastatic bladder cancer [1]. MIBC accounts for 20% of bladder cancer [2] and is associated with a high recurrence rate and metastasis potential. Additionally, 20–30% of patients initially diagnosed with NMIBC may progress to MIBC over time [3].

Radical cystectomy (RC) is the standard treatment for MIBC based on the guidelines of the Japanese Urological Association [4], National Comprehensive Cancer Network [5], and European Association of Urology [6]. Perioperative chemotherapy is recommended to improve survival outcomes. Randomized trials have demonstrated that cisplatin-based chemotherapy prolongs overall survival (OS) in patients with MIBC [7, 8]. Meta-analysis of adjuvant chemotherapy (AC) has not shown definitive OS benefit; however, some high-risk groups have exhibited improved survival [9, 10]. Thus, AC administration is often at the physician’s discretion. In Japan, an analysis of a multi-institutional database for perioperative chemotherapy in patients with UC conducted in 2008 discovered that 183 received perioperative chemotherapy out of 601 patients who had cT2–T4aN0M0 MIBC [11]. Additionally, the efficacy of perioperative chemotherapy has been described in the guidelines published in 2019; however, recommendations for a specific chemotherapy regimen have not been established [4]. Since 2019, the perioperative treatment strategy for MIBC has evolved, and the current treatment status, including specific regimens and duration, needs to be updated to identify the current challenges in perioperative care and provide fundamental information for future clinical trials and studies.

This study aimed to assess contemporary patient characteristics and perioperative treatments in UC in Japan. Specifically, we focused on bladder cancer, the most common type of UC, to clarify the actual perioperative treatment for MIBC and identify factors associated with receiving NAC using a hospital-based claims database.

##  Patients and methods

### Database

This retrospective, observational study used the hospital-based claims database from Medical Data Vision Co. Ltd. (MDV). The MDV database includes anonymized data from approximately 48 million patients collected from approximately 490 acute care hospitals, covering approximately 28% of all Diagnosis Procedure Combination hospitals in Japan [12]. The database includes insurance claims and discharge summary data collected from inpatients and outpatients, including basic information, diagnosis, prescription, medical practice, and laboratory test results.

### Patients

This study included patients with MIBC identified in the MDV database. The MDV database is a de-identified, hospital-based claims database covering April 2008 to July 2021, and participating hospitals contributed data for varying calendar periods. Patients were selected if they had at least one claim indicating UC recorded during January 1, 2012, to December 31, 2020 (case-identification window used to determine eligibility, not limited to the timing of initial diagnosis or surgery).

Cancer diagnoses were based on the International Classification of Diseases, 10th Revision (ICD-10) code “C.” The primary cancer site was identified using ICD-10 codes recorded in the month of initial cancer diagnosis. Bladder cancer cases were identified using the ICD-10 code “C67,” and only patients for whom the bladder was the primary site were included in the study. Patients with MIBC were defined as those who underwent RC, as identified by the codes in the receipt computer processing system in Japan: 150403310, 150407510, 150403410, 150407610, 150403510, 150407710, 150403610, 150403710, 150403810, 150200610, 150245910, 150246010, and 150246110 (Supplementary Table S1). Primary sites other than the bladder were classified based on ICD-10 codes for cancer diagnosed in the same month as the primary bladder cancer diagnosis.

### Variables

The patients’ demographic information, including age at primary bladder cancer diagnosis, height, weight, body mass index, Brinkman index as a smoking history, Eastern Cooperative Oncology Group (ECOG) performance status (PS; Supplementary Table S2) at the date of hospital admission for RC, and hospital size and type where RC was performed, were collected and analyzed. The size of the hospital was investigated based on the number of beds, and the type of hospital was determined based on whether it was designated as a cancer hospital. The clinical staging of bladder cancer was determined following the TNM Classification of Malignant Tumors of the Union for International Cancer Control (Supplementary Table S3). Clinical stage II or earlier was defined by ≤ T2, N0, M0, whereas clinical stage III or advanced stage was defined by ≥ T3. Comorbidities were classified into 17 categories based on the Charlson Comorbidity Index (CCI) using the corresponding ICD-10 codes recorded before and during the month of the initial bladder cancer diagnosis (Supplementary Table S4) [13, 14]. Laboratory data included estimated glomerular filtration rate (eGFR) (mL/min/1.73 m^2^) as an indicator of renal function. For patients who underwent NAC, the eGFR values were collected immediately before the first day of NAC. For those who did not receive NAC, the eGFR value was collected immediately before the admission date for RC. Treatment types were classified as follows: (1) NAC followed by RC (NAC group); (2) RC followed by AC (AC group); (3) a combination of NAC, RC, and AC (NAC + AC group); and (4) RC alone (RC-only group).

### Perioperative chemotherapy regimen

NAC was defined as chemotherapy started within 180 days before the date of RC, based on a previous study [15]. AC was defined as chemotherapy that began within 90 days of RC [16]. If anticancer agents were administered on the day before or on the same day as the platinum-based agent, they were considered to be part of the same regimen. Based on previous studies, the following seven regimens were investigated [9, 10, 17, 18].

- GC: gemcitabine + cisplatin.
- GCarbo: gemcitabine + carboplatin.
- M-VAC: methotrexate + vinblastine + doxorubicin + cisplatin.
- PGC: paclitaxel + gemcitabine + cisplatin.
- PGCarbo: paclitaxel + gemcitabine + carboplatin.
- M-VEC: methotrexate + vinblastine + epirubicin + cisplatin.
- CMV: cisplatin + methotrexate + vinblastine.

The number of courses was determined by counting the number of administrations of platinum-based agents. Continuous courses were defined as the administration of anticancer drugs without a gap of 90 days or more between doses [19].

### Statistical analysis

Patient characteristics are summarized as n (%) for categorical variables and as medians with interquartile ranges (IQR) for continuous variables. Missing or unrecorded values are retained as an explicit “unknown” category. To identify factors associated with receiving NAC, we compared the characteristics of patients who received NAC (NAC group) to those who did not (no-NAC group: AC group and RC-only group). Patients who underwent NAC and AC (NAC+AC group) were excluded. For categorical variables, comparisons were conducted using Pearson's chi-square test and Fisher's exact test. Continuous variables were assessed using the Mann–Whitney U test. To assess the robustness of the cohort definition, two sensitivity analyses were performed. The first restricted the cohort to patients with a recorded clinical stage ≥ cT2, and the second excluded patients whose clinical stage was unknown. All analyses of perioperative chemotherapy utilization and patient characteristics were repeated for these restricted cohorts using the same statistical procedures as in the primary analysis.

To further explore factors potentially associated with the administration of NAC, we conducted an exploratory multivariable logistic regression analysis. The dependent variable was receipt of NAC (NAC and NAC+AC, vs AC and RC-only, unlike univariable comparisons). Covariates included age, sex, CCI, BMI, clinical stage, hospital type, hospital size, and calendar year of surgery (treated as a continuous variable). We assessed multicollinearity using variance inflation factors (VIFs); all VIFs were ≤ 2, indicating minimal multicollinearity among covariates. ECOG-PS and eGFR were unavailable in the database and, therefore, excluded. Missing values were retained as an explicit “unknown” category to minimize exclusion bias. Results are presented as odds ratios with 95% confidence intervals. All statistical analyses were performed using SAS v. 9.4 (SAS Institute Inc., Cary, NC, USA), and statistical significance was set at *P* < 0.05.

### Ethical Considerations

The Ethics Committee of Keio University Faculty of Pharmacy approved this study (Approval No.: 240313–3).

## Results

**Fig. 1:**
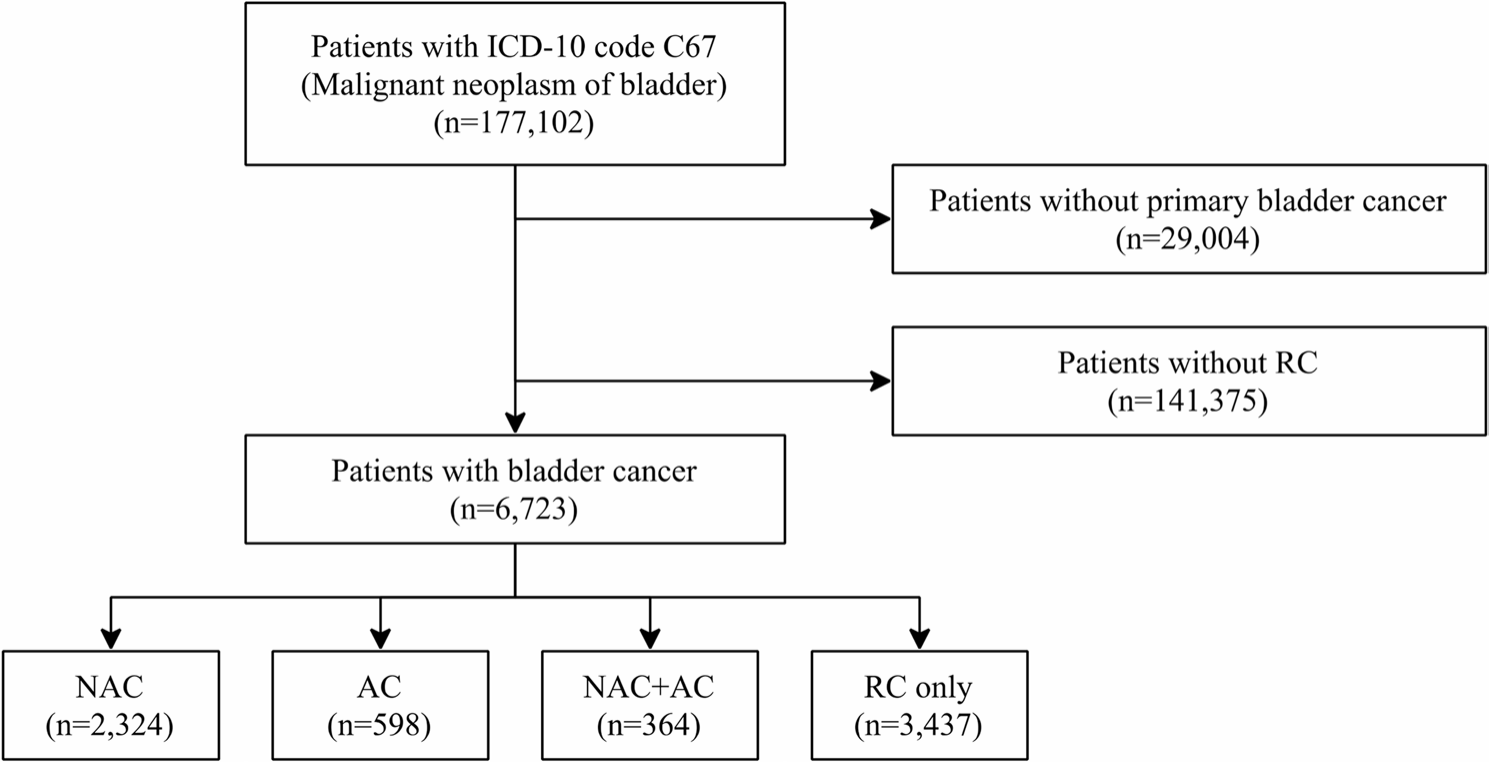
Patient selection process. *RC* Radical cystectomy, *NAC* Neoadjuvant chemotherapy, *AC* Adjuvant chemotherapy

Among 177,102 patients with ICD-10 code C67 (malignant neoplasm of the bladder), 6,723 were included after excluding those with suspected cancer diagnoses. Among these, 2,324 were in the NAC group, 598 in the AC group, 364 in the NAC + AC group, and 3,437 in the RC-only group (Fig. 1).

Table 1 summarizes patient characteristics. The median age at diagnosis was 71 years (IQR, 65–76), and 5,279 (78.5%) were men. The median observation period was 52 months (IQR 29–82 months). Regarding clinical staging, 2,516 patients (37.4%) were classified as clinical stage II or earlier, 1,806 (26.9%) had clinical stage III or more advanced, and 2,401 (35.7%) had an unknown clinical stage.

**Table 1:** Patient characteristics

Variables	All patients *n* = 6,723
Age at diagnosis, median (IQR)	71 (65–76) (*n* = 6,723)
Age at diagnosis, n (%)	
< 60	736 (10.9)
60–69	2,204 (32.8)
70–79	2,957 (44.0)
≥ 80	826 (12.3)
Sex, n (%)	
Women	1,444 (21.5)
Men	5,279 (78.5)
Height (cm), median (IQR)	163 (157–168) (*n* = 6,594)
Weight (kg), median (IQR)	60.0 (52.7–67.8) (*n* = 6,594)
BMI, median (IQR)	22.9 (20.7–25.0) (*n* = 6,594)
BMI, n (%)	
Underweight (< 18.5)	596 (8.9)
Normal weight (18.5–24.9)	4,335 (64.5)
Obese (≥ 25)	1,663 (24.7)
Unknown	129 (1.9)
CCI, median (IQR)	2 (2–3) (*n* = 6,723)
CCI, n (%)	
2	4,577 (68.1)
3	1,071 (15.9)
4	471 (7.0)
≥ 5	604 (9.0)
Smoking index, median (IQR)	205 (0–800) (*n* = 6,025)
Smoking index, n (%)	
0	2,705 (40.2)
1–399	614 (9.1)
400–799	1,122 (16.7)
800–1,199	988 (14.7)
≥ 1,200	596 (8.9)
Unknown	698 (10.4)
Primary or Recurrent, n (%)	
Primary	4,742 (70.5)
Recurrent	1,792 (26.7)
Unknown	189 (2.8)
Clinical Stage (UICC TNM), n (%)	
Ⅱ or less	2,516 (37.4)
Ⅲ or more	1,806 (26.9)
Unknown	2,401 (35.7)
Performance status, n (%)	
0	27 (0.4)
1	2 (0.03)
2	1 (0.01)
Unknown	6,693 (99.6)
Renal function (eGFR), median (IQR)	64.4 (50.7–77.3) (*n* = 717)
Renal function (eGFR), n (%)	
≥ 90.0	68 (1.0)
60.0–89.9	362 (5.4)
45.0–59.9	167 (2.5)
30.0–44.9	82 (1.2)
15.0–29.9	27 (0.4)
< 15.0	11 (0.2)
Unknown	6,006 (89.3)
Multiple primary sites, n (%)	
Ureter	251 (3.7)
Prostate	143 (2.1)
Renal pelvis	75 (1.1)
Colon	48 (0.7)
Gastric	28 (0.4)
Urethra	27 (0.4)
Kidney	23 (0.3)
Rectum	21 (0.3)
Others	287 (4.3)
Hospitals size based on number of beds, n (%)	
≤ 199	100 (1.5)
200–499	3,349 (49.8)
≥ 500	3,274 (48.7)
Hospital type, n (%)	
Designated cancer hospital	5,759 (85.7)
Non-designated cancer hospital	964 (14.3)
Observation period (months), median (IQR)	52 (29–82) (*n* = 6,723)
Days from diagnosis to surgery, median (IQR)	117 (71–189) (*n* = 6,723)
Surgical approach, n (%)	
Open	5,309 (79.0)
Laparoscopic	807 (12.0)
Robot-assisted	607 (9.0)
Perioperative chemotherapy, n (%)	
NAC	2,324 (34.6)
AC	598 (8.9)
NAC + AC	364 (5.4)
RC-only	3,437 (51.1)

*IQR* Interquartile range, *BMI*, Body mass index,* CCI* Charlson Comorbidity Index, *UICC* The Union for International Cancer Control, *TNM* TNM Classification, *eGFR* Estimated glomerular filtration rate, *RC* Radical cystectomy, *NAC* Neoadjuvant chemotherapy, *AC* Adjuvant chemotherapy

Supplementary Table S5 details patient characteristics for each treatment group. Among all patients, RC without perioperative chemotherapy (RC-only group) was the most common, accounting for 51.1%. The median age at diagnosis was 70 (IQR, 64–74) years in the NAC group, 69 (IQR, 64–74) years in the AC group, 68 (IQR, 62–72) years in the NAC + AC group, and 72 (IQR, 66–78) years in the RC-only group. Compared with the other (NAC: 6.9%, AC: 3.3%, NAC + AC: 1.9%) groups, the RC-only group had the largest proportion of patients aged 80 years or older (18.6%).

### Factors associated with receiving NAC

To identify the factors associated with NAC, we compared patient characteristics between the NAC and no-NAC groups (AC and RC-only) (Table 2). The median age at diagnosis in the NAC group (70 years [IQR: 64–74]) was significantly lower than that in the no-NAC group (72 years [IQR: 66–77]) (*P* < 0.0001). Both groups had similar sex distributions, with men comprising approximately 78% of patients in each group (*P* = 0.6332). The median CCI was 2 (IQR 2–3) in both groups, but significantly more patients with a CCI of 2 received NAC than those with a CCI of 3 or higher (*P* < 0.0001). The proportion of patients without prior treatment was higher in the NAC group (73.7%) than in the no-NAC group (68.1%; *P* < 0.0001). A greater proportion of patients in the NAC group were classified as clinical stage III or advanced (29.1% vs. 24.0%; *P* = 0.0033). The eGFR was also significantly higher in the NAC group (*P* = 0.0081). Hospital size was associated with NAC administration. More patients in the NAC group were treated at hospitals with ≥ 500 beds than in the no-NAC group (54.8% vs. 45.0%, *P* < 0.0001). A similar trend was observed for hospital type (designated cancer hospitals: 90.0% vs. 82.9%, *P* < 0.0001).

**Table 2:** Patients characteristics with or without receiving NAC

Variables	NAC (*n* = 2,324)	No NAC (*n* = 4,035)	*P*-value
Age at diagnosis, median (IQR)	70 (64–74) (*n* = 2,324)	72 (66–77) (*n* = 4,035)	< 0.0001
Age at diagnosis, n (%)			
< 60	290 (12.5)	383 (9.5)	
60–69	842 (36.2)	1,203 (29.8)	
70–79	1,032 (44.4)	1,790 (44.4)	
≥ 80 years	160 (6.9)	659 (16.3)	
Sex, n (%)			0.6332
Women	513 (22.1)	870 (21.6)	
Men	1,811 (77.9)	3,165 (78.4)	
Height (cm), median (IQR)	163 (158–168) (*n* = 2,285)	162 (156–168) (*n* = 3,946)	< 0.0001
Weight (kg), median (IQR)	60.6 (53.2–68.0) (*n* = 2,285)	59.8 (52.0–67.4) (*n* = 3,946)	0.0044
BMI, median (IQR)	22.8 (20.9–25.0) (*n* = 2,285)	22.9 (20.6–25.1) (*n* = 3,946)	0.9583
BMI, n (%)			
Underweight (< 18.5)	205 (8.8)	366 (9.1)	
Normal weight (18.5–24.9)	1,516 (65.2)	2,574 (63.8)	
Obese (≥ 25)	564 (24.3)	1,006 (24.9)	
Unknown	39 (1.7)	89 (2.2)	
CCI, median (IQR)	2 (2–3) (*n* = 2,324)	2 (2–3) (*n* = 4,035)	
CCI, n (%)			
2	1,676 (72.1)	2,633 (65.3)	< 0.0001
≥ 3	648 (27.9)	1,402 (34.7)	
Smoking index, median (IQR)	300 (0–800) (*n* = 2,085)	160 (0–800) (*n* = 3,607)	0.0068
Smoking index, n (%)			
0	889 (38.3)	1,696 (42.0)	
1–399	217 (9.3)	356 (8.8)	
400–799	410 (17.6)	634 (15.7)	
800–1,199	366 (15.7)	562 (13.9)	
≥ 1,200	203 (8.7)	359 (8.9)	
Unknown	239 (10.3)	428 (10.6)	
Primary or Recurrent, n (%)			< 0.0001
Primary	1,712 (73.7)	2,749 (68.1)	
Recurrent	551 (23.7)	1,159 (28.7)	
Unknown	61 (2.6)	127 (3.1)	
cStage (UICC TNM), n (%)			0.0033
Ⅱ or less	881 (37.9)	1,527 (37.8)	
Ⅲ or more	677 (29.1)	968 (24.0)	
Unknown	766 (33.0)	1,540 (38.2)	
Performance status, n (%)			1.0000
0	3 (0.1)	24 (0.6)	
1	0 (0.0)	2 (0.05)	
2	0 (0.0)	1 (0.02)	
Unknown	2,321 (99.9)	4,008 (99.3)	
Renal function (eGFR), median (IQR)	66.5 (53.1–78.3) (*n* = 301)	62.8 (46.6–76.1) (*n* = 356)	0.0081
Renal function (eGFR), n (%)			
≥ 90.0	33 (1.4)	30 (0.7)	
60.0–89.9	158 (6.8)	171 (4.2)	
45.0–59.9	77 (3.3)	79 (2.0)	
30.0–44.9	29 (1.2)	43 (1.1)	
15.0–29.9	3 (0.1)	23 (0.6)	
< 15.0	1 (0.04)	10 (0.2)	
Unknown	2,023 (87.0)	3,679 (91.2)	
Multiple primary sites, n (%)			< 0.0001
Yes	209 (9.0)	545 (13.5)	
No	2,115 (91.0)	3,490 (86.5)	
Hospitals size based on number of beds, n (%)			< 0.0001
≤ 199	18 (0.8)	80 (2.0)	
200–499	1,032 (44.4)	2,139 (53.0)	
≥ 500	1,274 (54.8)	1,816 (45.0)	
Hospital type, n (%)			< 0.0001
Designated cancer hospital	2,091 (90.0)	3,346 (82.9)	
Non-designated cancer hospital	233 (10.0)	689 (17.1)	
Observation period (months), median (IQR)	41 (24–66) (*n* = 2,324)	61 (34–87) (*n* = 4,035)	―
Days from diagnosis to surgery, median (IQR)	145 (116–192) (*n* = 2,324)	84 (55–189) (*n* = 4,035)	―
Surgical approach, n (%)			―
Open	1,679 (72.2)	3,329 (82.5)	
Laparoscopic	350 (15.1)	423 (10.5)	
Robot-assisted	295 (12.7)	283 (7.0)	

*IQR* Interquartile range, *BMI* Body mass index, *CCI* Charlson Comorbidity Index, *UICC* The Union for International Cancer Control, *TNM* TNM Classification, *eGFR* Estimated glomerular filtration rate, *NAC* Neoadjuvant chemotherapy

In an exploratory multivariable logistic regression analysis, younger age, more advanced clinical stage, treatment at designated cancer hospitals, treatment at large hospitals, and later calendar year of surgery were positively associated with NAC administration, whereas higher comorbidity burden and higher BMI were negatively associated (Supplementary Table S8). The overall direction and magnitude of these associations were consistent with the univariable findings.

### Sensitivity analyses

The results of the sensitivity analyses were consistent with those of the main analysis. When restricted to patients with recorded stage ≥ cT2, the proportion receiving NAC was 38.6%, similar to the overall rate of 34.6%. Excluding patients with unknown stage yielded comparable findings (36.0% NAC). The patient characteristics associated with NAC use showed no material changes in either restricted cohort (Supplementary Table S7), supporting the robustness of the study findings. The exploratory multivariable logistic regression analysis with ≥ cT2 and the stage-known cohort showed similar trends to the main analysis (Supplementary Table S8).

### Temporal trends in perioperative chemotherapy use

Supplementary Table S9 summarizes annual proportions of patients receiving NAC, AC, NAC + AC, or RC-only from 2008 to 2021. NAC utilization increased steadily over time (*P* for trend < 0.0001, derived from a multivariable logistic regression model treating year as a continuous variable, as summarized in Supplementary Table S8), whereas AC use remained comparatively stable. NAC use temporarily decreased in 2020 (41.2%) but rebounded in 2021 (48.1%).

### Perioperative chemotherapy regimen

Table 3 summarizes chemotherapy regimens and cycle number for NAC and AC. The median number of chemotherapy cycles among all patients receiving NAC was 2 (IQR, 2–3). The most common regimen was GC, which was administered to 1,846 patients (79.4%), followed by GCarbo in 354 patients (15.2%). A subset of 71 patients (3.1%) initially started GC treatment and then switched to GCarbo. In contrast, the median number of courses in patients who underwent AC was 2 (IQR, 2–3). GC was the most frequently administered regimen (461 patients, 77.1%), followed by GCarbo (114 patients, 19.1%). Supplementary Table S6 presents the results for patients who received NAC and AC.

**Table 3:** Chemotherapy regimens of patients who received NAC (*n* = 2,324) and AC (*n* = 598)

Regimen	NAC (*n* = 2,324)		AC (*n* = 598)	
	*n* (%)	No. of cycles, median (IQR)	*n* (%)	No. of cycles, median (IQR)
GC	1,846 (79.4)	2 (2–3)	461 (77.1)	2 (2–3)
GCarbo	354 (15.2)	2 (2–3)	114 (19.1)	3 (2–4)
M-VAC	18 (0.8)	3 (2–3)	10 (1.7)	2 (2–3)
PGC	13 (0.6)	2 (2–3)	1 (0.2)	―
PGCarbo	4 (0.2)	2.5 (2–4)	1 (0.2)	―
M-VEC	―	―	1 (0.2)	―
GC–GCarbo	71 (3.1)	2(2–3)	8 (1.3)	3.5 (2–5.5)
GC–M-VAC	7 (0.3)	2(1.5–2)	1 (0.2)	―
GC–M-VEC	1 (0.04)	―	―	―
GCarbo–GC	6 (0.3)	1.5(1–2)	―	―
GCarbo–M-VAC	1 (0.04)	―	―	―
GC–GCarbo–GC	―	―	1 (0.2)	―
GCarbo–GC –GCarbo	2 (0.09)	―	―	―
M-VAC–GC	1 (0.04)	―	―	―

*IQR* Interquartile range, *NAC* Neoadjuvant chemotherapy, *AC * Adjuvant chemotherapy,* GC* gemcitabine + cisplatin, *GCarbo* gemcitabine + carboplatin, *M-VAC* methotrexate + vinblastine + doxorubicin + cisplatin, *PGC* paclitaxel + gemcitabine + cisplatin, *PGCarbo* paclitaxel + gemcitabine + carboplatin, *M-VEC* methotrexate + vinblastine + epirubicin + cisplatin

## Discussion

We investigated the perioperative treatment of patients with MIBC and identified factors associated with NAC administration using a hospital-based claims database, providing important insight into the current clinical practice. We discovered that 35% of patients with MIBC received NAC, indicating an increasing trend in NAC use over time. Several factors, including age and clinical stage, were associated with NAC administration.

### Characteristics of patients with MIBC

Based on the National Cancer Center’s Hospital-based Cancer Registries [20], among 18,108 patients diagnosed with bladder cancer in Japan between 2014 and 2015, more than half were in clinical stage I, 20% in stage II, and 10% in stage III. The clinical stages distribution in our study was consistent with these data, supporting the generalizability of our results. In this study, 4,742 patients (70.5%) were diagnosed with primary bladder cancer, 1,792 patients (26.7%) with recurrent bladder cancer, and 189 (2.8%) with unknown primary bladder cancer. The Japanese Urological Association guidelines (2nd edition) published in 2015 [21], recommend RC for patients with recurrent Ta or T1 bladder cancer following transurethral resection of bladder tumor and Bacillus Calmette–Guérin intravesical therapy. Consistent with these guidelines, patients with primary and recurrent bladder cancers commonly underwent RC.

Among the patients with MIBC who underwent RC in our study, 2,516 patients (37.4%) had clinical stage II or earlier disease (≤ T2, N0, M0), 1,806 patients (26.9%) had clinical stage III or more advanced disease (≥ T3), and 2,401 (35.7%) had unknown staging. In a previous study [11] of 205 patients who underwent RC, 149 (72.7%) were classified as clinical T2 (cT2), whereas 56 (27.3%) were classified as T3–T4a, indicating a higher proportion of patients with T2 disease among these patients, which is consistent with our findings. The median age at diagnosis of patients with MIBC in this study was 71 years (IQR, 65–76 years), and 78.5% were men. These patient characteristics were consistent with those reported in a previous study in Japan [11].

The increasing use of NAC observed in our cohort was statistically significant (*P* for trend < 0.0001), as summarized in Supplementary Table S8 and S9. The proportion of patients receiving NAC rose steadily from 2008 to 2021, reaching nearly half of all RC cases by the end of the period. A temporary dip in 2020 and rebound in 2021 likely reflected short-term disruptions related to the COVID-19 pandemic. These findings illustrate a sustained shift toward the broader adoption of perioperative chemotherapy for MIBC in Japan during the past decade. In the previous study, the use of perioperative chemotherapy has increased since 2002 [11]. In patients who underwent RC, the rate of NAC increased from 12% to 34.6%, and the rate of AC increased from 8% to 8.9%. This trend may be attributed to the evidence from a randomized controlled trial and systematic review published in 2003, which demonstrated that cisplatin-based NAC improved OS in patients with MIBC. These findings were incorporated into the Japanese guidelines in 2009 [22]. Furthermore, an increasing trend in perioperative chemotherapy for MIBC has been observed in the United States [23. 24]. NAC contributes to survival benefits [7], and NAC is recommended in almost all clinical guidelines worldwide [23]. In the latest Japanese guidelines [24] issued in 2023 based on a systematic review and meta-analysis [25], AC is weakly recommended for patients who have not received cisplatin-based NAC, indicating a need for the accumulation of robust evidence. Because this study covered a fourteen-year observational period (2008–2021), temporal differences in treatment practice should be considered when interpreting our results. The increasing use of NAC observed during this period likely reflects evolving guideline recommendations and broader acceptance of perioperative chemotherapy in Japan. Importantly, our dataset precedes the introduction of immune checkpoint inhibitors (ICIs) for perioperative management; therefore, the results represent pre-ICI practice and may not fully capture current therapeutic strategies.

### Factors associated with NAC administration

The median age at diagnosis was significantly lower in the NAC group than in the no-NAC group. Additionally, the eGFR was significantly higher in the NAC group, and patients with a lower CCI were more likely to receive NAC. These trends are consistent with those of previous studies [23. 24]. The efficacy of NAC for UC in older adult patients is comparable to that in younger patients [26, 27]; however, older patients have multiple comorbidities and tend to have decreased renal function [28]. These physical conditions increase the likelihood of ineligibility for cisplatin-based chemotherapy [29, 30], thereby preventing perioperative chemotherapy [31]. Moreover, insufficient evidence regarding NAC in patients with renal impairment who are ineligible for cisplatin may further contribute to its lower utilization in older patients.

Regarding clinical staging, significantly more patients with stage III or more advanced disease received NAC, a trend consistent with previous studies [32, 33]. Based on the Japanese Urological Association Guidelines (3rd edition) [4], the standard treatment for stages II and III bladder cancer is RC with or without perioperative chemotherapy (NAC and/or AC) or bladder preservation therapy. Some observational studies have reported no significant survival benefit from NAC in patients with cT2N0M0 bladder cancer compared with those with cT3‒4aN0M0 [34]. This finding may have influenced physicians’ clinical decisions and contributed to the low NAC usage rate in patients with clinical stage II or earlier disease stages. Comparing the size of the treatment facilities, a significantly larger number of patients in the NAC group were treated in hospitals with ≥ 500 beds and designated cancer hospitals. These trends are consistent with those of previous studies [32, 33], indicating that hospitals with more experience in treating patients with MIBC are more likely to adhere to the established treatment guidelines [35–37].

In the exploratory multivariable analysis, several factors were independently associated with the receipt of NAC. Younger age, more advanced clinical stage, treatment at designated cancer hospitals, treatment at large hospitals, and later calendar year were positively associated with NAC administration, whereas higher comorbidity burden and higher BMI were negatively associated. These findings suggest that both patient fitness and institutional capability influenced the likelihood of receiving NAC, in line with previous real-world reports of treatment selection in MIBC. The significant effect of calendar year further confirms the temporal increase in NAC use demonstrated in Supplementary Table S9.

### Adjuvant chemotherapy adoption and intensified perioperative treatment (NAC + AC)

In our cohort, AC was administered to 598 patients (8.9%), indicating selective use in clinical practice. Among those who received NAC, 364 patients (5.4% of the total cohort) also received AC (NAC + AC), representing intensified perioperative management. Compared with patients who received NAC only, those who received additional AC were slightly younger (median 68 vs. 70 years) and more frequently had clinical stage III or more advanced disease (44% vs. 29%) (Supplementary Table S5). These differences suggest that sequential therapy was selectively used in individuals with high-risk disease or favorable postoperative recovery, rather than as a uniform strategy.

Although detailed pathological information, such as surgical margin status and tumor location, was unavailable in the MDV database, these unmeasured factors are known to influence postoperative treatment decisions and may partly explain the observed pattern of AC administration. Similar risk-adapted use of intensified perioperative chemotherapy has been reported in prior studies of locally aggressive disease [38, 39].

### Perioperative chemotherapy regimen for MIBC in Japan

The most common regimen for NAC and AC was gemcitabine plus cisplatin. In contrast, 20 of 33 patients who underwent NAC received M-VAC, the most common regimen, in 2008 [11]. This finding suggests that the perioperative chemotherapy regimens for MIBC have changed. Phase III clinical trials published in 2000 and 2005 for metastatic bladder cancer demonstrated that GC offered survival outcomes comparable with those of M-VAC with fewer side effects [40, 41]. Consequently, GC have been approved for bladder cancer in Japan [42]. Data from randomized studies remain limited; however, the results from several phase II trials and retrospective studies have shown that NAC for GC achieves a pathological complete response rate comparable to that of M-VAC [43–45]. This finding may explain the increased rate of GC observed in this study. GCarbo was the second common regimen in the NAC, AC, and NAC + AC groups, following GC therapy. Compared with data on the efficacy of GC, those on the efficacy of carboplatin-based chemotherapy for MIBC are limited. However, studies published in 2020 for metastatic bladder cancer and in 2022 for MIBC reported that NAC with GCarbo achieved a pathological complete response rate comparable to that of GC [46, 47]. Thus, GCarbo therapy can be considered an alternative to GC therapy in patients ineligible for cisplatin.

Most chemotherapy regimens involve a median of two cycles. A previous study [48] revealed that patients with MIBC receiving one to two NAC cycles had a lower response rates than those receiving three to four cycles. Whether the administration of one or two cycles was planned or due to deviations from the regimen remains unclear. However, further investigation into the optimal number of NAC cycles is essential to ensure that patients with MIBC fully benefit from NAC.

### Strengths and limitations

This study is the first to investigate the perioperative MIBC treatment in Japan using a large hospital-based claims database, clarifying perioperative chemotherapy regimens and cycle numbers. The strength of this study is that the patient distribution trends were consistent with prior registries, suggesting a highly representative of patients with MIBC in Japan [11]. Thus, these results reflect the characteristics and perioperative treatment of patients with MIBC in Japan. Furthermore, patients with MIBC were defined by the RC procedure codes alongside ICD-10 codes for bladder cancer. This approach made it unlikely that patients without MIBC would be included in the analysis, and instances of false-positive cases were considered rare. An inherent methodological consideration is the operational definition of MIBC in this study. Because the MDV database lacks direct pathological information, we identified MIBC cases using ICD-10 diagnostic codes for bladder cancer (C67) in combination with procedure codes for RC, following the approach used in previous Japanese database studies. The stage variable reflects clinical staging as recorded in hospital claims, not pathological findings. Although this definition is expected to capture most MIBC cases treated with RC, it may also include a small number of patients who underwent cystectomy for other indications, such as BCG-refractory NMIBC. To evaluate the potential influence of such misclassification, we conducted sensitivity analyses restricting the cohort to patients with documented ≥ cT2 disease and, separately, excluding those with unknown stage. The results were consistent with the main findings, confirming that the overall patterns of NAC utilization and associated patient characteristics remained robust across definitions.

This study had some limitations. First, the positive predictive value of cancer definitions based on ICD-10 codes is approximately 80% based on a previous validation study [49]. Consequently, this study excluded patients who underwent RC but did not have an appropriately assigned diagnosis of bladder cancer in the database and those whose primary cancer was not bladder cancer. This exclusion could have led to an underestimation of the number of eligible patients. Second, data on some parameters such as clinical stage, ECOG PS, and eGFR were missing from MDV database. This omission may limit the accurate characterization of the patients’ backgrounds, and unmeasured factors may have influenced the decision to administer perioperative chemotherapy. In addition, information on variant histologies according to the WHO 2022 classification was unavailable in the MDV database. Variant subtypes such as micropapillary, plasmacytoid, and squamous/glandular differentiation are recognized prognostic factors and may influence perioperative decision-making, including the likelihood of receiving NAC or AC [50–53]. Because these data were not captured, unmeasured heterogeneity in tumor biology cannot be excluded. Finally, tracking patients’ medical and prescription histories across hospitals was impossible because hospital-based claims database was used. This limitation may have led to incomplete data collection on comorbidities and treatments, particularly in patients who received NAC or AC at a different institution and underwent surgery elsewhere. Consequently, the rates of perioperative chemotherapy reported here may underestimate actual utilization in Japan. While the MDV database covers a large proportion of Diagnosis Procedure Combination hospitals and broadly reflects real-world practice, our findings should be interpreted as representing treatment patterns within the MDV network rather than the entire national population. Detailed oncologic outcomes such as pathologic response, recurrence, and survival were also unavailable in the MDV database; therefore, this study focused on process measures, specifically, perioperative treatment patterns, rather than clinical outcomes. Moreover, the full observation window (2008–2021) encompassed substantial changes in guideline recommendations, but it did not include the introduction of novel agents such as ICIs. These temporal shifts could have influenced perioperative treatment decisions and regimen selection, and our findings should therefore be interpreted as reflecting pre-ICI practice patterns in Japan. These findings provide important baseline data reflecting the treatment decisions currently made in the Japanese clinical environment.

## Conclusion

This study provides a comprehensive overview of perioperative treatment trends for Japanese patients with MIBC and revealed that the utilization rate of perioperative chemotherapy increased annually. Patient characteristics and treatment facilities were factors influencing NAC administration.

## Supplementary Information

Supplementary material 1.

## Data Availability

The data that support the findings of this study are not openly available for reasons of sensitivity but are available from the corresponding author upon reasonable request.
