# Supplementary material for: Survey of perioperative treatment in muscle-invasive bladder cancer using Japanese hospital-based claims database
Source: BMC Urol. 2025 Nov 24;25:309. doi: 10.1186/s12894-025-01995-1 (PMC12751781; doi:10.1186/s12894-025-01995-1)
Supplement: Supplementary file 1 — Supplementary material 1. [file 12894_2025_1995_MOESM1_ESM.docx]

Survey of perioperative treatment in muscle-invasive bladder cancer using Japanese hospital-based claims database

*BMC Urology*

Ayumi Yamazawa, Masami Tsuchiya, Shungo Imai, Keisuke Ikegami, Hayato Kizaki, and Satoko Hori

Division of Drug Informatics, Keio University Faculty of Pharmacy. 1-5-30 Shibakoen, Minato-ku, Tokyo 105-8512, Japan

Corresponding Author

Email: [satokoh@keio.jp](mailto:satokoh@keio.jp)

**Table S1.** Radical cystectomy codes in the receipt computer processing system in Japan

| Receipt code | Name of medical practice |
| --- | --- |
| 150403310 | Laparoscopic surgery for malignant bladder tumors  (Total cystectomy, no urinary diversion using intestinal tract) |
| 150407510 | Laparoscopic surgery for malignant bladder tumors  (No urinary diversion using intestinal tract, general rule 18) |
| 150403410 | Laparoscopic surgery for malignant bladder tumors  (Total cystectomy, urinary diversion using ileal conduit) |
| 150407610 | Laparoscopic surgery for malignant bladder tumors  (Urinary diversion using ileal conduit, general rule 18) |
| 150403510 | Laparoscopic surgery for malignant bladder tumors  (Total cystectomy, urinary diversion using bladder substitution) |
| 150407710 | Laparoscopic surgery for malignant bladder tumors  (Urinary diversion using bladder substitution, general rule 18) |
| 150403610 | Laparoscopic minimum incision surgery for malignant bladder tumors  (Total cystectomy, no urinary diversion using intestinal tract) |
| 150403710 | Laparoscopic minimum incision surgery for malignant bladder tumors  (Total cystectomy, urinary diversion using ileal conduit) |
| 150403810 | Laparoscopic minimum incision surgery for malignant bladder tumors  (Total cystectomy, urinary diversion using bladder substitution) |
| 150200610 | Surgery for malignant bladder tumors  (Total cystectomy [No urinary diversion using intestinal tract]) |
| 150245910 | Surgery for malignant bladder tumors  (Total cystectomy [Urinary diversion using ureterosigmoidostom]) |
| 150246010 | Surgery for malignant bladder tumors  (Total cystectomy [Urinary diversion using ileal conduit]) |
| 150246110 | Surgery for malignant bladder tumors  (Total cystectomy [Urinary diversion using bladder substitution]) |

[Reference]

Hokatsu Shien Kakari, Iryoka, Health Insurance Bureau, Ministry of Health and Welfare. [Reiwa 6 nendo shindangun bunrui (DPC) denshi tensuhyo.] (in Japanese). Available at: <https://www.mhlw.go.jp/stf/seisakunitsuite/bunya/0000198757_00008.html>. accessed 15 Dec 2024.

**Table S2.** ECOG Performance Status Scale

| GRADE | ECOG PERFORMANCE STATUS |
| --- | --- |
| 0 | Fully active, able to carry on all pre-disease performance without restriction |
| 1 | Restricted in physically strenuous activity but ambulatory and able to carry out work of a light or sedentary nature, such as light house work, office work |
| 2 | Ambulatory and capable of all selfcare but unable to carry out any work activities; up and about more than 50% of waking hours |
| 3 | Capable of only limited selfcare; confined to bed or chair more than 50% of waking hours |
| 4 | Completely disabled; cannot carry on any selfcare; totally confined to bed or chair |
| 5 | Dead |

[Reference]

Oken MM, Creech RH, Tormey DC, Horton J, Davis TE, McFadden ET, Carbone PP. Toxicity and response criteria of the Eastern Cooperative Oncology Group. *Am J Clin Oncol*. 1982 Dec;5(6):649-655. PMID: 7165009.

**Table S3.** UICC TNM classification

7th edition

| Stage | T | N | M |
| --- | --- | --- | --- |
| 0a | Ta | N0 | M0 |
| 0is | Tis | N0 | M0 |
| Ⅰ | T1 | N0 | M0 |
| Ⅱ | T2a, T2b | N0 | M0 |
| Ⅲ | T3a, T3b | N0 | M0 |
|  | T4a | N0 | M0 |
| Ⅳ | T4b | N0 | M0 |
|  | Any T | N1, N2, N3 | M0 |
|  | Any T | Any N | M1 |

8th edition

| Stage | T | N | M |
| --- | --- | --- | --- |
| 0a | Ta | N0 | M0 |
| 0is | Tis | N0 | M0 |
| Ⅰ | T1 | N0 | M0 |
| Ⅱ | T2 | N0 | M0 |
| ⅢA | T3, T4a | N0 | M0 |
|  | T1, T2 T3, T4a | N1 | M0 |
| ⅢB | T1, T2 T3, T4a | N2, N3 | M0 |
| ⅣA | T4b | Any N | M0 |
|  | Any T | Any N | M1a |
| ⅣB | Any T | Any N | M1b |

[References]

・ Sobin LH, Gospodarowicz MK, Wittekind C. International Union Against Cancer (UICC) TNM classification of malignant tumours. 7. New York, Wiley-Liss, 2010.

・ J.D. Brierley, M.K. Gospodarowicz, C. Wittekind. TNM Classification of Malignant Tumors, 8th Edition, Oxford, Wiley Blackwell, UK, 2016.

Table S4. ICD-10 coding algorithms of CCI

| Comorbidities | ICD-10 |
| --- | --- |
| Myocardial infarction | I21.x, I22.x, I25.2 |
| Congestive heart failure | I09.9, I11.0, I13.0, I13.2, I25.5, I42.0, I42.5–I42.9, I43.x, I50.x, P29.0 |
| Peripheral vascular disease | I70.x, I71.x, I73.1, I73.8, I73.9, I77.1, I79.0, I79.2, K55.1, K55.8, K55.9, Z95.8, Z95.9 |
| Cerebrovascular disease | G45.x, G46.x, H34.0, I60.x–I69.x |
| Dementia | F00.x–F03.x, F05.1, G30.x, G31.1 |
| Chronic pulmonary disease | I27.8, I27.9, J40.x–J47.x, J60.x–J67.x, J68.4, J70.1, J70.3 |
| Rheumatic disease | M05.x, M06.x, M31.5, M32.x–M34.x, M35.1, M35.3, M36.0 |
| Peptic ulcer disease | K25.x–K28.x |
| Mild liver disease | B18.x, K70.0–K70.3, K70.9, K71.3–K71.5, K71.7, K73.x, K74.x, K76.0, K76.2–K76.4, K76.8, K76.9, Z94.4 |
| Diabetes without chronic complication | E10.0, E10.1, E10.9, E11.0, E11.1, E11.9, E12.0, E12.1, E12.9, E13.0, E13.1, E13.9, E14.0, E14.1, E14.9 |
| Diabetes with chronic complication | E10.2–E10.5, E10.7, E11.2–E11.5, E11.7, E12.2–E12.5, E12.7, E13.2–E13.5, E13.7, E14.2–E14.5, E14.7 |
| Hemiplegia or paraplegia | G04.1, G11.4, G80.1, G80.2, G81.x, G82.x, G83.0–G83.4, G83.9 |
| Renal disease | I12.0, I13.1, N03.2–N03.7, N05.2–N05.7, N18.x, N19.x, N25.0, Z49.0–Z49.2, Z94.0, Z99.2 |
| Any malignancy, including lymphoma and leukemia, except malignant neoplasm of skin | C00.x–C26.x, C30.x–C34.x, C37.x–C41.x, C43.x–C58.x, C60.x–C76.x, C81.x–C88.x, C90.x–C97.x |
| Moderate or severe liver disease | I85.0, I85.9, I86.4, I98.2, K70.4, K71.1, K72.1, K72.9, K76.5, K76.6, K76.7 |
| Metastatic solid tumor | C77.x–C80.x |
| AIDS/HIV | B20.x–B22.x, B24.x |

Abbreviations: AIDS/HIV, acquired immunodeficiency syndrome/human immunodeficiency virus; CCI, Charlson Comorbidity Index; ICD-10, International Classification of Diseases, 10th Revision

**Table S5.** Patient background by treatment group

| Variables | NAC  (n=2,324) | AC  (n=598) | NAC+AC  (n=364) | RC only  (n=3,437) |
| --- | --- | --- | --- | --- |
| Age at diagnosis,  median (IQR) | 70 (64–74)  (n=2,324) | 69 (64–74)  (n=598) | 68 (62–72)  (n=364) | 72 (66–78)  (n=3,437) |
| Age at diagnosis, n (%) |  |  |  |  |
| < 60 | 290 (12.5) | 83 (13.9) | 63 (17.3) | 300 (8.7) |
| 60–69 | 842 (36.2) | 242 (40.5) | 159 (43.7) | 961 (28.0) |
| 70–79 | 1,032 (44.4) | 253 (42.3) | 135 (37.1) | 1,537 (44.7) |
| ≥ 80 | 160 (6.9) | 20 (3.3) | 7 (1.9) | 639 (18.6) |
| Sex, n (%) |  |  |  |  |
| Women | 513 (22.1) | 120 (20.1) | 61 (16.8) | 750 (21.8) |
| Men | 1,811 (77.9) | 478 (79.9) | 303 (83.2) | 2,687 (78.2) |
| Height (cm), median (IQR) | 163 (158–168)  (n=2,285) | 164 (158–169)  (n=583) | 164 (159–170)  (n=363) | 162 (156–167)  (n=3,363) |
| Weight (kg), median (IQR) | 60.6  (53.2–68.0)  (n=2,285) | 61.2  (53.7–68.9)  (n=583) | 61.7  (54.7–69.2)  (n=363) | 59.5  (51.8–67.1)  (n=3,363) |
| BMI, median (IQR) | 22.8  (20.9–25.0)  (n=2,285) | 23.0  (20.8–25.0)  (n=583) | 22.9  (20.8–25.1)  (n=363) | 22.9  (20.6–25.1)  (n=3,363) |
| BMI, n (%) |  |  |  |  |
| Underweight (< 18.5) | 205 (8.8) | 48 (8.0) | 25 (6.9) | 318 (9.3) |
| Normal weight  (18.5–24.9) | 1,516 (65.2) | 393 (65.7) | 245 (67.3) | 2,181 (63.5) |
| Obese (≥ 25) | 564 (24.3) | 142 (23.7) | 93 (25.5) | 864 (25.1) |
| Unknown | 39 (1.7) | 15 (2.5) | 1 (0.3) | 74 (2.2) |
| CCI, median (IQR) | 2 (2–3)  (n=2,324) | 2 (2–3)  (n=598) | 2 (2–3)  (n=364) | 2 (2–3)  (n=3,437) |
| CCI, n (%) |  |  |  |  |
| 2 | 1,676 (72.1) | 430 (71.9) | 268 (73.6) | 2,203 (64.1) |
| 3 | 341 (14.7) | 83 (13.9) | 39 (10.7) | 608 (17.7) |
| 4 | 125 (5.4) | 42 (7.0) | 12 (3.3) | 292 (8.5) |
| ≥ 5 | 182 (7.8) | 43 (7.2) | 45 (12.4) | 334 (9.7) |
|  |  |  |  |  |
| Smoking index,  median (IQR) | 300 (0–800)  (n=2,085) | 270 (0–800)  (n=528) | 420 (0–820)  (n=333) | 124 (0–800)  (n=3,079) |
| Smoking index, n (%) |  |  |  |  |
| 0 | 889 (38.3) | 226 (37.8) | 120 (33.0) | 1,470 (42.8) |
| 1–399 | 217 (9.3) | 58 (9.7) | 41 (11.3) | 298 (8.7) |
| 400–799 | 410 (17.6) | 98 (16.4) | 78 (21.4) | 536 (15.6) |
| 800–1,199 | 366 (15.7) | 86 (14.4) | 60 (16.5) | 476 (13.8) |
| ≥ 1,200 | 203 (8.7) | 60 (10.0) | 34 (9.3) | 299 (8.7) |
| Unknown | 239 (10.3) | 70 (11.7) | 31 (8.5) | 358 (10.4) |
| Primary or Recurrent, n (%) |  |  |  |  |
| Primary | 1,712 (73.7) | 448 (74.9) | 281 (77.2) | 2,301 (66.9) |
| Recurrent | 551 (23.7) | 143 (23.9) | 82 (22.5) | 1,016 (29.6) |
| Unknown | 61 (2.6) | 7 (1.2) | 1 (0.3) | 120 (3.5) |
| Clinical Stage (UICC TNM), n (%) |  |  |  |  |
| Ⅱ or less | 881 (37.9) | 140 (23.4) | 108 (29.7) | 1,387 (40.4) |
| Ⅲ or more | 677 (29.1) | 271 (45.3) | 161 (44.2) | 697 (20.3) |
| Unknown | 766 (33.0) | 187 (31.3) | 95 (26.1) | 1,353 (39.4) |
| Performance status, n (%) |  |  |  |  |
| 0 | 3 (0.1) | 6 (1.0) | 0 (0.0) | 18 (0.5) |
| 1 | 0 (0.0) | 0 (0.0) | 0 (0.0) | 2 (0.06) |
| 2 | 0 (0.0) | 0 (0.0) | 0 (0.0) | 1 (0.03) |
| Unknown | 2,321 (99.9) | 592 (99.0) | 364 (100.0) | 3,416 (99.4) |
| Renal function (eGFR),  median (IQR) | 66.5 (53.1–78.3) (n=301) | 59.9 (46.9–78.2) (n=50) | 62.8  (51.4–77.8) (n=60) | 63.0  (46.4–76.0) (n=306) |
| Renal function (eGFR),  n (%) |  |  |  |  |
| ≥ 90.0 | 33 (1.4) | 3 (0.5) | 5 (1.4) | 27 (0.8) |
| 60.0–89.9 | 158 (6.8) | 22 (3.7) | 33 (9.1) | 149 (4.3) |
| 45.0–59.9 | 77 (3.3) | 15 (2.5) | 11 (3.0) | 64 (1.9) |
| 30.0–44.9 | 29 (1.2) | 5 (0.8) | 10 (2.7) | 38 (1.1) |
| 15.0–29.9 | 3 (0.1) | 5 (0.8) | 1 (0.3) | 18 (0.5) |
| < 15.0 | 1 (0.04) | 0 (0.0) | 0 (0.0) | 10 (0.3) |
| Unknown | 2,023 (87.0) | 548 (91.6) | 304 (83.5) | 3,131 (91.1) |
| Multiple primary sites, n (%) |  |  |  |  |
| Yes | 209 (9.0) | 69 (11.5) | 49 (13.5) | 476 (13.8) |
| No | 2,115 (91.0) | 529 (88.5) | 315 (86.5) | 2,961 (86.2) |
| Hospitals size based on number of beds, n (%) |  |  |  |  |
| ≤ 199 | 18 (0.8) | 8 (1.3) | 2 (0.5) | 72 (2.1) |
| 200–499 | 1,032 (44.4) | 342 (57.2) | 178 (48.9) | 1,797 (52.3) |
| ≥ 500 | 1,274 (54.8) | 248 (41.5) | 184 (50.5) | 1,568 (45.6) |
| Hospital type, n (%) |  |  |  |  |
| Designated cancer hospital | 2,091 (90.0) | 490 (81.9) | 322 (88.5) | 2,856 (83.1) |
| Non-designated cancer hospital | 233 (10.0) | 108 (18.1) | 42 (11.5) | 581 (16.9) |
| Observation period (months), median (IQR) | 41 (24–66)  (n=2,324) | 67 (36–94)  (n=598) | 53 (28–77)  (n=364) | 60 (33–87)  (n=3,437) |
| Days from diagnosis to  surgery, median (IQR) | 145 (116–192)  (n=2,324) | 73 (50–110)  (n=598) | 138  (108–171)  (n=364) | 86 (56–218)  (n=3,437) |
| Surgical approach, n (%) |  |  |  |  |
| Open | 1,679 (72.2) | 538 (90.0) | 301 (82.7) | 2,791 (81.2) |
| Laparoscopic | 350 (15.1) | 39 (6.5) | 34 (9.3) | 384 (11.2) |
| Robot-assisted | 295 (12.7) | 21 (3.5) | 29 (8.0) | 262 (7.6) |

IQR, Interquartile range; BMI, Body mass index; CCI, Charlson Comorbidity Index; UICC, The Union for International Cancer Control; TNM, TNM Classification; eGFR, estimated glomerular filtration rate; RC, Radical cystectomy; NAC, Neoadjuvant chemotherapy; AC, Adjuvant chemotherapy

**Table S6.** Chemotherapy regimens of patients who received NAC and AC (n=364)

| Regimen | | n (%) | NAC No. of cycles, median (IQR) | AC No. of cycles, median (IQR) |
| --- | --- | --- | --- | --- |
| NAC | AC |  |  |  |
| GC | GC | 248 (68.1) | 2 (2–2) | 2 (2–2) |
| GC | GCarbo | 28 (7.7) | 2 (2–2.5) | 2 (2–3) |
| GC | M-VAC | 16 (4.4) | 3 (2–3) | 3 (2–3) |
| GC | M-VEC | 1 (0.3) | ― | ― |
| GC | PGC | 2 (0.5) | ― | ― |
| GC | CMV | 1 (0.3) | ― | ― |
| GCarbo | GC | 8 (2.2) | 2 (2–2.5) | 2 (1.5–3) |
| GCarbo | GCarbo | 35 (9.6) | 2 (2–3) | 2 (2–3) |
| M-VAC | GC | 1 (0.3) | ― | ― |
| PGC | M-VAC | 1 (0.3) | ― | ― |
| PGC | PGC | 2 (0.5) | ― | ― |
| GC | GC–GCarbo | 7 (1.9) | ― | ― |
| GC | GC–GCarbo–GC | 1 (0.3) | ― | ― |
| GC | GCarbo–GC | 1 (0.3) | ― | ― |
| GC–GCarbo | GC | 3 (0.8) | ― | ― |
| GC–GCarbo | GCarbo | 5 (1.3) | ― | ― |
| GC–GCarbo | GCarbo–GC | 1 (0.3) | ― | ― |
| GCarbo–GC | GC | 1 (0.3) | ― | ― |
| M-VAC–GC | GC | 1 (0.3) | ― | ― |
| M-VAC–GCa | GCarbo | 1 (0.3) | ― | ― |

IQR, Interquartile range; NAC, Neoadjuvant chemotherapy; AC, Adjuvant chemotherapy; GC, gemcitabine + cisplatin; GCarbo, GCa, gemcitabine + carboplatin; M-VAC, methotrexate + vinblastine + doxorubicin + cisplatin; PGC, paclitaxel + gemcitabine + cisplatin; CMV, cisplatin + methotrexate + vinblastine; M-VEC, methotrexate + vinblastine + epirubicin + cisplatin

**Table S7.** Baseline characteristics and perioperative treatment patterns in the main cohort and sensitivity cohorts (≥ clinical T2 cohort and stage-known cohort).

| Variables | All patients  n=6,723 | ≥ clinical T2 cohort  n=3,645 | Stage-known cohort  n=4,322 |
| --- | --- | --- | --- |
| Age at diagnosis, median (IQR) | 71 (65–76)  (n=6,723) | 71 (65–76)  (n=3,645) | 71 (65–76)  (n=4,322) |
| Age at diagnosis, n (%) |  |  |  |
| < 60 | 736 (10.9) | 422 (11.6) | 512 (11.9) |
| 60–69 | 2,204 (32.8) | 1,185 (32.5) | 1,413 (32.7) |
| 70–79 | 2,957 (44.0) | 1,585 (43.5) | 1,886 (43.6) |
| ≥ 80 | 826 (12.3) | 453 (12.4) | 511 (11.8) |
| Sex, n (%) |  |  |  |
| Women | 1,444 (21.5) | 824 (22.6) | 950 (22.0) |
| Men | 5,279 (78.5) | 2,821 (77.4) | 3,372 (78.0) |
| BMI, n (%) |  |  |  |
| Underweight (< 18.5) | 596 (8.9) | 343 (9.4) | 390 (9.0) |
| Normal weight (18.5–24.9) | 4,335 (64.5) | 2,363 (64.8) | 2,797 (64.7) |
| Obese (≥ 25) | 1,663 (24.7) | 900 (24.7) | 1,087 (25.2) |
| Unknown | 129 (1.9) | 39 (1.1) | 48 (1.1) |
| CCI, median (IQR) | 2 (2–3)  (n=6,723) | 2 (2–3)  (n=3,645) | 2 (2–3)  (n=4,322) |
| CCI, n (%) |  |  |  |
| 2 | 4,577 (68.1) | 2,513 (68.9) | 2,997 (69.3) |
| 3 | 1,071 (15.9) | 581 (15.9) | 678 (15.7) |
| 4 | 471 (7.0) | 233 (6.4) | 280 (6.5) |
| ≥ 5 | 604 (9.0) | 318 (8.8) | 367 (8.5) |
| Clinical Stage (UICC TNM), n (%) |  |  |  |
| Ⅱ or less | 2,516 (37.4) | 1,686 (46.2) | 2,516 (58.2) |
| Ⅲ or more | 1,806 (26.9) | 1,777 (48.8) | 1,806 (41.8) |
| Unknown | 2,401 (35.7) | 182 (5.0) | 0 (0) |
| Hospitals size based on number of beds, n (%) |  |  |  |
| ≤ 199 | 100 (1.5) | 57 (1.6) | 62 (1.4) |
| 200–499 | 3,349 (49.8) | 1,861 (51.0) | 2,242 (51.9) |
| ≥ 500 | 3,274 (48.7) | 1,727 (47.4) | 2,018 (46.7) |
| Hospital type, n (%) |  |  |  |
| Designated cancer hospital | 5,759 (85.7) | 3,097 (85.0) | 3,652 (84.5) |
| Non-designated cancer hospital | 964 (14.3) | 548 (15.0) | 670 (15.5) |
| Surgical approach, n (%) |  |  |  |
| Open | 5,309 (79.0) | 2,935 (80.5) | 3,466 (80.2) |
| Laparoscopic | 807 (12.0) | 398 (10.9) | 482 (11.2) |
| Robot-assisted | 607 (9.0) | 312 (8.6) | 374 (8.6) |
| Perioperative chemotherapy, n (%) |  |  |  |
| NAC | 2,324 (34.6) | 1,405 (38.6) | 1,556 (36.0) |
| AC | 598 (8.9) | 380 (10.4) | 410 (9.5) |
| NAC+AC | 364 (5.4) | 253 (6.9) | 268 (6.2) |
| RC-only | 3,437 (51.1) | 1,607 (44.1) | 2,088 (48.3) |

IQR, Interquartile range; BMI, Body mass index; CCI, Charlson Comorbidity Index; UICC, The Union for International Cancer Control; TNM, TNM Classification; RC, Radical cystectomy; NAC, Neoadjuvant chemotherapy; AC, Adjuvant chemotherapy

**Table S8.** Multivariable logistic regression analysis of factors associated with receipt of neoadjuvant chemotherapy in the main and sensitivity cohorts.

| Variables | All patients  n=6,594 | ≥ clinical T2 cohort  n=3,606 | Stage-known cohort  n=4,274 |
| --- | --- | --- | --- |
|  | aOR (95% CI) | aOR (95% CI) | aOR (95% CI) |
| Age (per 10-year increase) | 0.679 (0.639–0.722) | 0.647 (0.595–0.703) | 0.900 (0.770–1.052) |
| Sex |  |  |  |
| Women (ref) | 1.000 | 1.000 | 1.000 |
| Men | 0.964 (0.850–1.093) | 0.980 (0.830–1.159) | 0.900 (0.770–1.052) |
| BMI |  |  |  |
| Underweight (< 18.5) | 0.904 (0.751–1.088) | 0.817 (0.640–1.042) | 0.836 (0.664–1.052) |
| Normal weight (18.5–24.9, ref) | 1.000 | 1.000 | 1.000 |
| Obese (≥ 25) | 0.854 (0.756–0.964) | 0.838 (0.711–0.987) | 0.874 (0.752–1.016) |
| Missing | 1.272 (0.844–1.916) | 0.760 (0.348–1.657) | 0.878 (0.431–1.788) |
| CCI |  |  |  |
| 2 (ref) | 1.000 | 1.000 | 1.000 |
| 3 | 0.770 (0.667–0.890) | 0.817 (0.640–1.042) | 0.797 (0.666–0.954) |
| 4 | 0.595 (0.480–0.737) | 0.838 (0.711–0.987) | 0.480 (0.360–0.639) |
| ≥5 | 0.779 (0.649–0.935) | 0.760 (0.348–1.657) | 0.871 (0.692–1.097) |
| Clinical Stage |  |  |  |
| I­–II (ref) | 1.000 | 1.000 | 1.000 |
| ≥III | 1.416 (1.246–1.610) | 1.093 (0.948–1.261) | 1.423 (1.249–1.620) |
| Unknown | 0.839 (0.744–0.947) | 0.811 (0.581–1.131) | - |
| Hospitals size |  |  |  |
| <500 (ref) | 1.000 | 1.000 | 1.000 |
| ≥500 | 1.187 (1.063–1.326) | 1.175 (1.011–1.364) | 1.222 (1.064–1.403) |
| Hospital Type |  |  |  |
| Non-designated cancer hospital (ref) | 1.000 | 1.000 | 1.000 |
| Designated cancer hospital | 1.594 (1.349–1.883) | 1.888 (1.517–2.350) | 1.731 (1.415–2.117) |
|  |  |  |  |
| Year of surgery* (per 1-year increase) | 1.174 (1.151–1.197) | 1.209 (1.177–1.241) | 1.185 (1.156–1.214) |

IQR, Interquartile range; BMI, Body mass index; CCI, Charlson Comorbidity Index; UICC, The Union for International Cancer Control; TNM, TNM Classification; RC, Radical cystectomy; NAC, Neoadjuvant chemotherapy; AC, Adjuvant chemotherapy

*Year was modeled as a continuous variable (centered at 2019); its p-value is reported as the adjusted p for trend.

*Note:* Sample sizes shown in the column headers represent the total number of patients in each cohort. Because of missing data in the year of surgery, the number of patients included in the multivariable model was slightly smaller (complete-case analysis).

**Table S9.** Annual trends in perioperative chemotherapy use among patients undergoing radical cystectomy, 2008–2021.

| Year | Total | NAC  (n, %) | AC  (n, %) | NAC+AC  (n, %) | RC-only  (n, %) |
| --- | --- | --- | --- | --- | --- |
| 2008 | 21 | 0 (0) | 4 (19.0) | 0 (0) | 17 (81.0) |
| 2009 | 29 | 11 (37.9) | 5 (17.2) | 0 (0) | 13 (44.8) |
| 2010 | 110 | 18 (16.4) | 16 (14.5) | 5 (4.5) | 71 (64.5) |
| 2011 | 164 | 24 (14.6) | 34 (20.7) | 9 (5.5) | 97 (59.1) |
| 2012 | 231 | 41 (17.7) | 30 (13.0) | 7 (3.0) | 153 (66.2) |
| 2013 | 330 | 62 (18.8) | 55 (16.7) | 15 (4.5) | 198 (60.0) |
| 2014 | 559 | 125 (22.4) | 58 (10.4) | 29 (5.2) | 347 (62.1) |
| 2015 | 593 | 163 (27.5) | 65 (11.0) | 38 (6.4) | 327 (55.1) |
| 2016 | 687 | 234 (34.1) | 54 (7.9) | 52 (7.6) | 347 (50.5) |
| 2017 | 648 | 232 (35.8) | 57 (8.8) | 42 (6.5) | 317 (48.9) |
| 2018 | 883 | 351 (39.8) | 65 (7.4) | 44 (5.0) | 423 (47.9) |
| 2019 | 999 | 431 (43.1) | 61 (6.1) | 50 (5.0) | 457 (45.7) |
| 2020 | 1082 | 446 (41.2) | 79 (7.3) | 49 (4.5) | 508 (47.0) |
| 2021 | 387 | 186 (48.1) | 15 (3.9) | 24 (6.2) | 162 (41.9) |
| Total | 6,723 | 2,324 (34.6) | 598 (8.9) | 364 (5.4) | 3,437 (51.1) |
